# Caffeine consumption within British fencing athletes

**DOI:** 10.3389/fnut.2022.999847

**Published:** 2022-11-11

**Authors:** George Bowra Morris, Ralph Johannes Francisca Manders, Andrew Thomas Hulton

**Affiliations:** Department of Nutritional Sciences, Faculty of Health and Medical Sciences, University of Surrey, Guildford, United Kingdom

**Keywords:** caffeine, ergogenic aids, dietary habits, fencing, performance

## Abstract

The ergogenic effects of caffeine are well documented, yet despite the potential benefits of supplementation, there is a lack of understanding of caffeine habits and supplementation within fencing. British fencers (*n* = 136) completed a Web-based questionnaire, exploring self-reported caffeine consumption, reasons for use and education. Fencers (94.1%) habitually consumed caffeine, primarily due to the taste of the products (93.8%). Respondents ingested 183.4 ± 137.5 mg of caffeine daily, with a significant difference between age groups (*p* < 0.05). Many respondents (30.1%) consumed caffeine 60 mins prior/during fencing training and/or competition with the main reason highlighted as cognitive performance enhancement. Respondents ingested 140.8 ± 104.6 mg of caffeine during training/competition, mainly as energy drinks, bars, and powders. Education on caffeine supplementation was low (25.7%), with significant associations between age groups (*p* < 0.05). Evidence implies caffeine toxicity has been experienced by 35% of fencers, highlighting the need for education on caffeine consumption. To conclude there is evidence of caffeine supplementation in fencing, primarily to magnify cognitive performance. However, there is a requirement for targeted education on caffeine supplementation to fencers, so that negative side effects and potential anti-doping infringements can be avoided.

## Introduction

Fencing is classed as an open-skilled combat sport comprising three disciplines (the épée, the foil, and the saber). Performance in all disciplines is characterized by recurrent short bouts of high intensity exercise, dispersed within periods of lower intensity exercise (1). Fencing matches take place on a 14 m × 2 m strip, known as a “piste” with points scored following contact through the weapon, with the discipline specific target areas and by which fencer can have priority (2, 3). The winner of a fencing match is the first athlete to score five points during the preliminary pool matches (1 × 3 mins), or fifteen points (3 × 3 mins) during the direct elimination matches (3). A competition can be between 9 and 11 h, although effective fight time is greatly reduced (2, 4).

Literature has highlighted key psychological parameters as important determinants of fencing performance (2–5). Research have stated that performance, in fencing, is likely linked with cognitive processes such as perceptual processing, neuro-physiological characteristics, the need to anticipate opponent’s actions and specific stimulus-response relationships (2). Further, it is cognitive decisions and movements made within fractions of a second that govern fencing performance (5). These findings are consistent with those devised by Bottoms et al. (4), who suggested that an athlete’s reaction time in response to their opponent’s attack is a principal determinant of successful fencing performance. Therefore, the evidence base suggests that cognitive variables, specifically a fencer’s reaction/response time in retaliation to their opponent’s actions, are central to a fencer’s success when competing. Implications may suggest that fencers could bolster performance through the enhancement of psychological parameters. Potential strategies for enhancement could include the utilization of an ergogenic aid, such as caffeine.

Caffeine (1,3,7-trimethylxanthine) is a legal psychoactive drug consumed by approximately 90% of adults daily (6). Following caffeine’s removal from the World Anti-Doping Agency (WADA) Prohibited List (2004), caffeine has been used as an ergogenic aid among athletes (4, 7, 8). The effects of caffeine in reducing fatigue, and enhancing wakefulness and alertness are well recognized (8–10). Specific research into the effects of caffeine’s ability to enhance fencing performance observed improvements in fencer’s reaction time (5), as well as significant reductions in perceived exertion and a tendency for fewer ‘misses’ during a skill test (4). Furthermore, research (11) demonstrated that neuromuscular fatigue is associated with decrements in reaction time in open-skilled combat sports, suggesting previous findings (4) could have practical implications for an improved fencing performance. However, negative side effects of caffeine consumption also exist. Accordingly, excessive caffeine consumption (>500–600 mg) is associated with cardiovascular symptoms including tachycardia and arrythmia, with complications arising in those predisposed to cardiovascular health conditions (12).

The primary mechanism of caffeine action remains inconclusive, although several theories have been proposed for the potential ergogenic effects of caffeine on cognitive and neuromuscular performance. Adenosine antagonism, resulting in the stimulation of the central nervous system (CNS) and subsequently causing a dampened pain perception, improved central drive and greater muscle fiber recruitment is a highlighted theoretical mechanism of action for potential ergogenic effects on cognitive performance (13). Furthermore, additional research (14) has suggested that caffeine has a direct effect on the CNS, affecting the perception of effort and the propagation of neural signals.

The prevalence of and reasons for caffeine consumption have previously been investigated in cyclists, track and field athletes and Ironman triathletes (7, 15). Additionally, Del Coso et al. (8) measured the caffeine concentration in 20,686 urine samples between 2004 and 2008 with elite athletes participating in a magnitude of sports included in the sample population. Findings included that three out of four athletes had consumed caffeine before or during sports competition, with endurance sports showing the highest urinary caffeine excretion post-competition. However, despite the highlighted potential benefits of caffeine supplementation on fencing performance, and the existing literature investigating caffeine and fencing performance (4, 5) there is a scarcity of knowledge regarding its use within this sporting population, such as consumption, reasons for use, and sources of education. Therefore, the aim of this study was to examine: (a) the prevalence and type of caffeinated products used by British fencers; (b) the reasons for caffeine consumption; and (c) the type and sources of information British fencers have received regarding caffeine usage in sport and fencing. Overall, the present study is novel and will aid in the understanding of the caffeine habits of British Fencing athletes. The authors hypothesize that; (a) elite level British fencers consume greater amounts of caffeine than non-elite level British fencers; (b) with a priority of enhancing fencing performance; and (c) following advice directed by sport nutritionists and literature-based research.

## Materials and methods

### Respondents

British fencers (*n* = 136) completed the Web-based questionnaire anonymously. The inclusion criteria were male and female, age 16 or over and currently training/competing. Participants were excluded from the present study if they were under the age of 16 years or not currently training/competing in the sport of fencing. Additionally, partially completed Web-based questionnaires were excluded from the final statistical analyses. Respondents read and completed the participant information and informed consent sheet providing informed consent. The study received a favorable ethical opinion from the institution’s Research Ethics Committee and was conducted in accordance with the Ethical Standards in Sport and Exercise Science Research: 2020 Update (16).

### Questionnaire development

Twenty- seven questions were separated into seven sections: participant information sheet; informed consent; respondent details; general caffeine consumption (outside of training/competition period); specific caffeine consumption (caffeine solely consumed in the 60-mins before/during fencing training and/or competition); perceived benefits and perceived side-effects of consumption; sources of information/advice regarding caffeine use.

Sections aimed to collect: demographic information on the study respondents; information on general caffeine consumption, including sources of caffeine, the amount and reasons for consumption; fencing specific information on caffeine consumption, such as sources of caffeine, the amount, reasons for consumption and whether or not caffeine is used as an ergogenic aid for both training and competition; data regarding the athletes perception of how caffeine helps or hinders their performance; information regarding whether respondents had received education/advice on the consumption of caffeine in fencing/general sport, and the sources of this information. Qualtrics*^XM^* software (Qualtrics*^XM^*, Dublin, Ireland) was used to build the Web-based questionnaire. The questionnaire was piloted using university staff, students, and general population (*n* = 8). Following the pilot work refinements were made to questions, ensuring clarity, coherence, and effective data collection.

### Questionnaire distribution

Data were collected between the 2nd of November and the 31st of December 2020. During this period, respondents were directed to the Web-based questionnaire via recruitment emails and social media posts/messages. Recruitment material was directed to fencing clubs, club leaders, and fencers. Emails, social media posts and messages contained information regarding the aims and purposes of the study, inclusion criteria and the Web-based questionnaire link. Corresponding to this, the British Fencing national governing body dispatched the same recruitment email to mailing lists including members of British Fencing’s Athlete Development Programme (ADP); and current “compete” members of British Fencing.

### Statistical analysis

Completed questionnaires were coded and entered into a data file using the IBM Statistical Package for the Social Sciences (SPSS v27) (Chicago, IL, USA) for subsequent analysis. Caffeine intake was determined through the conversion of popular household foodstuff measures/quantities via normative foodstuff values from a collection of external sources including published caffeine data; food composition database data; analytical reports; and manufacturer’s data (14, 17–24). Measures of centrality and spread are reported as means ± standard deviation (SD). All data were assessed for normality. Comparisons between sex and caffeine consumption were conducted using Mann Whitney *U* statistical tests. Kruskall Wallis statistical tests were used to compare caffeine consumption between fencing weapons; competition level; and age group. Statistical differences were followed up with *post hoc* testing. Chi-Square tests for association were used to compare frequency between sex; fencing weapon participated in; competition level; and age groups, on whether respondents had received education on caffeine consumption. Statistical significance was set at *p* < 0.05 for all analyses.

## Results

### Respondent demographic details

The response rate for the Web-based questionnaire was unknown because of the sampling strategy employed. The present study only collected data from 2.2% of the total number of fencers currently holding a compete membership in the United Kingdom (approximately 6,000 fencers from all abilities). However, the investigation did engage with 52.2% of the British fencers competing internationally, which was determined utilizing the British Fencing ranking schemes and data from the British Fencing Association (25). Respondent characteristics are displayed in Table 1. The sample size of the present investigation is larger than previous research (26) using a similar study design and investigating sport supplement usage within fencers.

**TABLE 1:** Characteristics of web-based questionnaire respondents (*n* = 136).

Characteristic/category	Overall number (%)
Sex	
Male	80 (58.8)
Female	56 (41.2)
Age Group	
U17	18 (13.2)
U20	33 (24.3)
U23	22 (16.2)
Senior	36 (26.5)
Veteran	27 (19.9)
Discipline/Weapon	
Épée	73 (53.7)
Foil	36 (26.5)
Sabre	27 (19.9)
Level of Competition	
Recreational	0 (0)
Club	5 (3.7)
County	8 (5.9)
Regional	5 (3.7)
National	22 (16.2)
International	96 (70.6)

Senior = ≥ 20 years of age; Veteran = ≥40 years of age.

### General caffeine consumption

Of respondents, 94.1% consumed caffeine containing products in their diet outside of fencing training and/or competition. Caffeinated products were consumed for varying reasons. Enjoyment of the taste of products was the most popular reason for consumption (Table 2), with other reasons including a perceived increased alertness and a reduction in fatigue.

**TABLE 2:** Reasons for general caffeinated product consumption.

Reason for caffeinated product consumption	Frequency (%)
Enjoyment of the taste	93.8%
Work/study purposes	46.9%
Social aspects	25.4%
Perceived health benefits	14.6%
Other	14.6%

**FIGURE 1:**
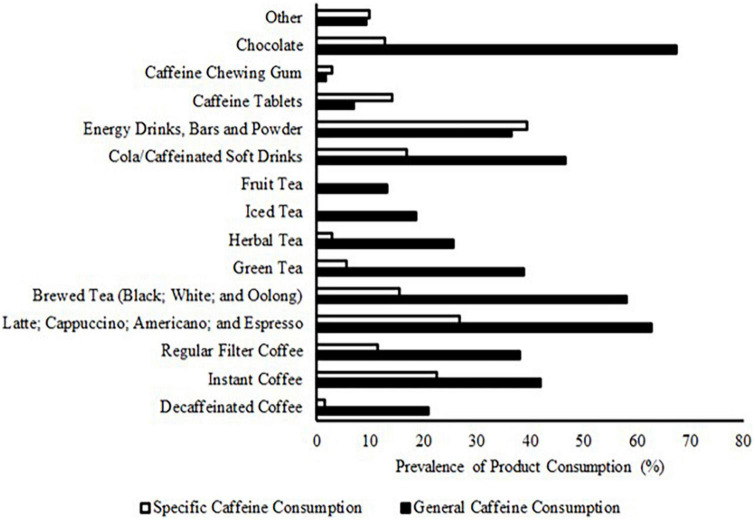
Prevalence of caffeinated product consumption (%) within British Fencing athletes during a typical 24-h period outside of fencing training/competition (general caffeine consumption) and before (1-h preceding)/during fencing training and/or competition (specific caffeine consumption).

Chocolate was the most consumed caffeine containing product, closely followed by coffee drink derivatives: Latte, cappuccino, americano, and espresso; instant; regular filter; and decaffeinated (Figure 1).

**FIGURE 2:**
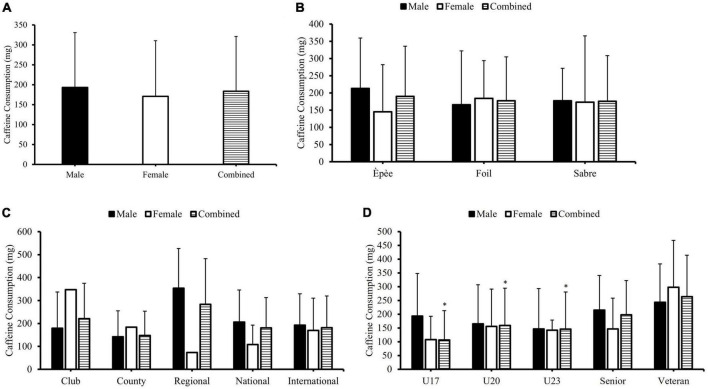
The differences in the typical caffeine consumption of British Fencing athletes, during the 24-h period outside of fencing training/competition (general caffeine consumption) when comparing sex **(A)**, fencing discipline participated in panel **(B)**, competition level **(C)**, and age group **(D)**, within British Fencing athletes. *A statistically significant difference (*p* < 0.05) in the combined general caffeine consumption in relation to the veteran age group.

Respondents ingested 183.4 ± 137.5 mg of caffeine during a typical 24-h period, outside of fencing training and/or competition, with no significant differences between sex (*p* ≥ 0.05; Figure 2A); fencing discipline (*p* ≥ 0.05; Figure 2B); and level of competition (*p* ≥ 0.05; Figure 2C) and the amount of caffeine consumed. A significant difference between age group (*p* < 0.01) and the amount of caffeine consumed during a typical 24-h period outside of fencing training and/or competition was identified. *Post hoc* statistical tests identified significant differences between the veteran age group and the U17, U20, and U23 age groups, whereby veteran participants consumed the largest quantity of caffeine (Figure 2D).

### Specific caffeine consumption

**FIGURE 3:**
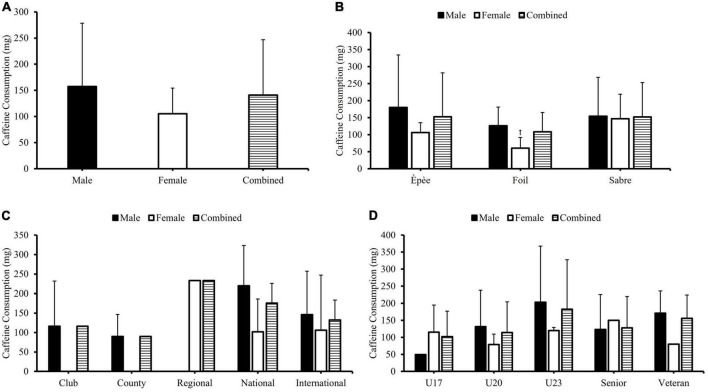
The differences in the typical caffeine consumption of British Fencing athletes, before (1-h preceding)/during fencing training and/or competition (specific caffeine consumption) when comparing sex **(A)**, fencing discipline participated in panel **(B)**, competition level **(C)**, and age group **(D)**, within British Fencing athletes. ^†^A statistically significant difference (*p* < 0.05) in the specific caffeine consumption of females, in relation to males, who trained/competed in the foil fencing discipline.

Respondents ingested 140.8 ± 104.6 mg of caffeine before/during fencing training and/or competition. There was no significant difference between any of the subgroups (Figure 3). However, there was a significant difference (*p* < 0.05) between the specific caffeine consumption of males and females who trained/competed within the foil fencing discipline, whereby females consumed less caffeine.

In total, 30.1% (*n* = 41) declared caffeine consumption prior to (60-mins before) and/or during fencing training and/or competition. Of those athletes, 84.9% stated that their main reason for the ingestion of caffeinated products was to potentially enhance performance (Table 3). Other reasons for caffeine use (6.8%) were associated with “increased alertness” and “decreased fatigue”. Most respondents revealed that caffeinated products were consumed solely before/during competition (50.7%). Caffeine was consumed before/during both training and competition by 45.2% of respondents, with 4.1% solely consuming caffeine before/during fencing training.

**TABLE 3:** Reasons for specific caffeinated product consumption.

Reason for caffeinated product consumption	Frequency (%)
Potentially enhance performance	84.9%
Enjoyment of the taste	64.4%
Perceived health benefits	13.7%
Work/study purposes	6.8%
Social aspects	5.5%
Other	6.8%

Energy drinks, bars and powders were the most consumed products before/during fencing training and/or competition, with 39.4% of respondents consuming this category of product. “Red Bull” and “Monster Energy” energy drinks were the most consumed products in this category. Unspecified “pre-workout” supplement drink mixes were also a popular product. Cola/caffeinated soft drinks were ingested by 16.9% of respondents, desired products included: “Coca-Cola zero sugar”; “Coca-Cola”; and “pepsi MAX”. Caffeine tablets (specifically “PRO PLUS”) were consumed by a higher percentage of respondents before/during fencing training and/or competition (14.1%), as was caffeine chewing gum (2.8%), compared to general caffeine consumption. Other products consumed that may contain caffeine (9.9%) included: “caffeine shots;” “caffeine gels;” and “chocolate covered coffee beans” (Figure 1).

### Perceived ergogenic effects of caffeine consumption

Of respondents consuming caffeine to potentially enhance performance (*n* = 62), 93.5% highlighted that effects associated with CNS stimulation, were beneficial to their performance at training and/or competition. Words that sum up the respondent’s reasons for consumption include focus, energized and alert. A reduced perception of fatigue was emphasized by 6.5% of respondents, following caffeine consumption with: “reduced feeling of fatigue and exertion;” and an ability to help “manage fatigue” highlighted.

### Perceived side effects of caffeine consumption

When questioned about unwanted side effects, 35% (*n* = 48) of respondents had experienced side effects following caffeine consumption. Of those respondents, 31% reported experiencing tremors, “jitters” or “shaking”. “Headaches” was also a common side effect, reported by 16.7% of the 48 respondents with 14.6% experiencing gastrointestinal distress.

### Education on caffeine

Overall, 25.7% of respondents reported receiving education on caffeine (Table 4). There was no significant association between: sex (*p* ≥ 0.05), fencing discipline (*p* ≥ 0.05) and competition level (*p* ≥ 0.05). However, there was a significant association between age and education on caffeine (*p* < 0.05). Of respondents who had received education on caffeine supplementation (*n* = 35), 48.6% had received this from sports coaches, 34.3% reported receiving education from a nutritionist, 20% highlighted academics and educational material as a source of information, 8.6% sought information from unspecified internet sources and 11.4% acquired advice from fellow athletes.

**TABLE 4:** A comparison between participant variables and the obtaining of education on caffeine supplementation.

Variable	Frequency of respondents who have received education (%)	Frequency of respondents who have not received education (%)
Sex		
Male	26.3	73.8
Female	25	75
Fencing discipline		
Èpèe	30.1	69.9
Sabre	11.1	88.9
Foil	27.8	72.2
Level of competition		
Recreational	–	–
Club	0	100
County	0	100
Regional	0	100
National	22.7	77.3
International	31.3	68.8
Age group		
U17	33.3	66.7
U20	33.3	66.7
U23	45.5	54.5
Senior	16.7	83.3
Veteran	7.4	92.6

U17, under 17 years of age; U20, under 20 years of age; Senior, over 20 years of age; veteran, aged 40 years of over.

## Discussion

The present study aimed to examine: (a) the prevalence and type of caffeinated products used by British fencers; (b) reasons for caffeine consumption; (c) and the type and sources of information received regarding caffeine usage in fencing. The key finding was that over half of respondents consumed caffeine in the hour preceding or during fencing training and/or competition, and of those 84.9% stated that this was to potentially enhance performance. Overall respondents received minimal education regarding caffeine supplementation, with sports coaches providing the most educational information. Habitual caffeine ingestion was also common among respondents, with respondents consuming higher doses than non-athletes (27).

As the present study determined that 30.1% of respondents regularly consume caffeine in the hour preceding or during a fencing performance, this finding is consistent with previous research investigating sport supplement usage within fencers (26). Therefore, the present study further supports the notion that fencers use caffeine as an ergogenic aid, to enhance training/competition performance. Moreover, the findings are also consistent with the prevalence of caffeine consumption reported in track and field athletes, but lower than that reported in cyclists (7). Perhaps this implies that caffeine supplementation strategies are increasingly prevailing and becoming an accepted nutritional strategy within fencing. This could be because of the high cognitive and neuromuscular demands of fencing, and the requirement for skill and perception maintenance over prolonged durations (2). A theory that is consistent with the amount, and perceived ergogenic effects. Further, an overwhelming majority (93.5%) highlighted perceived ergogenic effects linked to central nervous system stimulation, and the associated cognitive benefits, as perceived ergogenic effects - with increased alertness and a promotion of wakefulness being at the forefront.

There were no significant main differences between gender in caffeine consumption during the present study. This highlights that both males and females consume similar quantities of caffeine both habitually and specifically for use as an ergogenic aid. Moreover, these findings are again supported by Mata et al. (26) within their results that demonstrate no differences in the proportion of male and female fencers who consumed caffeine as an ergogenic aid. However, during the present study, there was a significant difference in specific caffeine consumption between gender within the foil fencing discipline, whereby males consumed more caffeine. Despite this difference, as this was not consistent throughout the general and specific caffeine consumption of all three disciplines it cannot be concluded that male and female fencers consume differing quantities of caffeine. Therefore, the present study supports previous reports (26).

There was no significant difference between the level of competition and the amount of specific caffeine consumption, which contradicts previous findings (7). It was concluded that a greater proportion of higher-level athletes and cyclists consumed caffeine to potentially enhance performance (7), which was implied to be a result of elite-populations receiving more education about the ergogenic effects of caffeine and are therefore more likely to adopt supplementation protocols (7). Overall, 25.7% of respondents received education on caffeine with no significant difference between competition level. Potential differences could be that fencing is classified as a progression sport by UK Sport (28). Therefore, receiving little funding compared to mainstream sports like cycling and athletics. Fencing will receive an investment from UK Sport, that is 6% of the total investment that cycling will receive for the same period, hence resulting in the National Governing Body (NGB) lacking the resources for widespread education programmes (2021–2025 period) (28). There was a significant association in age and having received education on caffeine. The U17, U20, and U23 age groups stated they had received education compared to senior and veteran fencers. The authors suggest this is a likely product of those age groups coordinating with the eligible ages for the ADP and the Diploma in Sporting Excellence (DiSE) programmes, existing “to support athletes in achieving Olympic success” offering additional educational opportunities (29, 30). This is further indicated through the sources of information received. Over one-third of respondents (34.3%) received education on caffeine from a nutritionist. Of those respondents, a number (*n* = 6) specifically mentioned “ADP” and “DiSE” as the source of the nutritionists’ advice. A large frequency of respondents received education from sports coaches (48.6%). This finding is not isolated to the present study, with previous investigations highlighting 48–50% of nutritional education being delivered by a “coach or trainer” (31, 32). Despite a wealth of knowledge and specialist skills related to sports practice, it is unlikely that specialist sports coaches possess the nutritional knowledge equivalent to a nutrition expert. Furthermore, it is likely that the nutritional knowledge base across sports coaches is highly variable.

Respondents habitually consumed 183.4 ± 137.5 mg of caffeine each day. Contrasting previous conclusions (27) suggesting that habitual intake of caffeine is ∼130 mg day^–1^ within the UK. This indicates that British fencers consume increased amounts of caffeine daily compared to non-athletic populations. Utilizing normative body mass (BM) data of fencers, general caffeine consumption within the present study was 2.5 ± 0.8 mg kg^–1^ (33). Thus, suggesting that British fencers are unlikely to experience blunted ergogenic effects due to their habituation, as previously suggested chronic intakes ∼3 mg kg^–1^ BM per day would not affect the acute ergogenic effect. Nevertheless, considerable variation exists in caffeine responsiveness between individuals (34). With such inter-individual variation, the relationship between caffeine habituation and performance is highly complex. The authors highlight the need for a pragmatic approach to caffeine supplementation in real-world athletes.

There was a significant difference between respondent age group and general caffeine consumption. Veteran age group fencers reported the highest mean general caffeine consumption, with the U17 age group reporting the least. This trend could be a result of social and cultural norms associated with caffeinated product consumption. Previously described as a brewed tea drinking nation, it is estimated that 81% of the UK population consumed brewed tea (35), although, reduced in younger age groups. Energy drinks, bars and powders were the most frequently consumed product before/during fencing training and/or competition. This is unsurprising given the potential practical issues associated with products habitually ingested, such as: consumption of large volumes of liquid; and issues in the quantification of caffeine intake (36). Respondents also reported consuming “pre workout” sports supplements. Alarmingly, various pre workout sports supplements have been found to contain banned substances. Namely, Cohen et al. (37) identified a methamphetamine analog in a mainstream dietary supplement. Therefore, athletes consuming this classification of product could be at increased risk of failing a drug test, due to ingesting a supplement tainted with a compound listed on the WADA prohibited list.

The side effects reported in the present study are consistent with that of current literature (12). Caffeine toxicity was reported in 35% of all respondents. Symptoms detailed were mostly indicators of general caffeine toxicity, ranging from: nervousness; irritability; gastrointestinal disturbances; arrhythmia; and tachycardia. Literature has suggested chronic exposure of >500–600 mg of caffeine per day poses a significant health risk and is regarded as “abuse”, which if sustained results in “caffeinism” (12). Many respondents in the current study reported symptoms of “caffeinism,” including restlessness; anxiety; irritability; muscle tremor; insomnia; headaches; cardiovascular symptoms; and gastrointestinal complaints (38). This is unsurprising given the maximal habitual caffeine intake identified within the current investigation was 628 mg. Education provision to fencers should include the potential side effects and issues associated with caffeine toxicity and chronic abuse. Moreover, education provision is particularly important for fencers predisposed to enhanced cardiovascular risks following caffeine consumption, i.e., excessive habitual caffeine consumers (>500–600 mg of caffeine per day), those consuming an acute high caffeine doses (>300 mg of caffeine), and fencers with pre-existing cardiovascular risk factors for adverse reactions (12). Furthermore, by outlining the requirement for targeted education delivery, this highlights the importance for preparticipation screening for cardiovascular risk factors that may predispose a fencer to adverse reactions to caffeine consumption.

The main limitation of the present study is the utilization of a retrospective, Web-based questionnaire design. Although such methods allowed for the collection of a larger sample size, when compared to conventional questionnaires, the study design was limited by a lack of consideration for the sample size and the resulting significance. Furthermore, sub-elite level fencers were underrepresented within the current study. This leads to caution when interpreting results for the influence of competition level. Therefore, future research should be conducted utilizing a stratified random sample of fencers, allowing for a more representative sample population, addressing the key limitation.

The findings of the present study highlight the use of caffeine supplementation for performance enhancement, within British Fencing athletes. Possible real-world practical applications include requirement for expansion of educational programmes to offer a wider range of advice and information to high-performance athletes; the implementation of coach education/continued professional development to further enhance the nutritional knowledge of sports coaches; and the need for further widespread education on anti-doping. In conclusion, the current evidence implies the ubiquitous use of caffeine supplementation among British Fencers. Results indicated that this was primarily for performance enhancement purposes to magnify cognitive performance. British Fencers also ingested high quantities of caffeine habitually. Overall, there is a necessity for education on caffeine supplementation directed toward athletes and coaches, and a requirement for future research to explore both the benefits and risks associated with long-term caffeine consumption within general and specific athletic populations.

## Data availability statement

The original contributions presented in the study are included in the article/Supplementary material, further inquiries can be directed to the corresponding author.

## Ethics statement

The studies involving human participants were reviewed and approved by Faculty of Health and Medical Sciences Research Committee, University of Surrey. Written informed consent from the participants’ legal guardian/next of kin was not required to participate in this study in accordance with the national legislation and the institutional requirements.

## Author contributions

AH and GM designed the study, with GM collecting the data. GM conducted data analysis and interpretation, drafted the manuscript, and received critical revisions from AH and RM. All authors contributed to the article and approved the submitted version.
