# Supplementary material for: Caffeine consumption within British fencing athletes
Source: Front Nutr. 2022 Nov 11;9:999847. doi: 10.3389/fnut.2022.999847 (PMC9691662; doi:10.3389/fnut.2022.999847)
Supplement: Supplementary file 2 [file Table_3.DOCX]

| Supplementary Table 1. The quantity of caffeine in commonly consumed foodstuffs | | |
| --- | --- | --- |
|  |  |  |
| Foodstuff(s) |  | Typical Caffeine Content per Serving (mg) |
|  |  |  |
| Coffee |  |  |
|  | Decaffeinated | 3 |
|  | Instant | 50 |
|  | Regular Filter | 124 |
|  | Latte | 60 |
|  | Cappuccino | 60 |
|  | Americano | 60 |
|  | Espresso | 75 |
| Tea |  |  |
|  | White/Black/Oolong | 36 |
|  | Green/Herbal | 0 |
| Other Drinks |  |  |
|  | Cola | 30 |
|  | Energy Drinks | 80 |
| Other Foodstuffs |  |  |
|  | Energy Bars/Powders | 80 |
|  | Caffeine Chewing Gum | 100 |
|  | Caffeine Tablets | 50 |
|  | Dark Chocolate | 80 (per 100 g) |
|  |  |  |
| Note: Normative caffeine values from a collection of external sources including published caffeine data, food composition databases, analytical reports, and data from manufacturers (Coca Cola Great Britain, 2020; Desbrow et al., 2007; Food Standards Agency, 2004; Healthspan, 2021; Nutritics, 2019; Pro Plus, 2021; Redbull, 2021; Spreit, 1995; Starbucks, 2021). | | |
