# Supplementary figures and images for: Caffeine consumption within British fencing athletes
Source: Front Nutr. 2022 Nov 11;9:999847. doi: 10.3389/fnut.2022.999847 (PMC9691662; doi:10.3389/fnut.2022.999847)

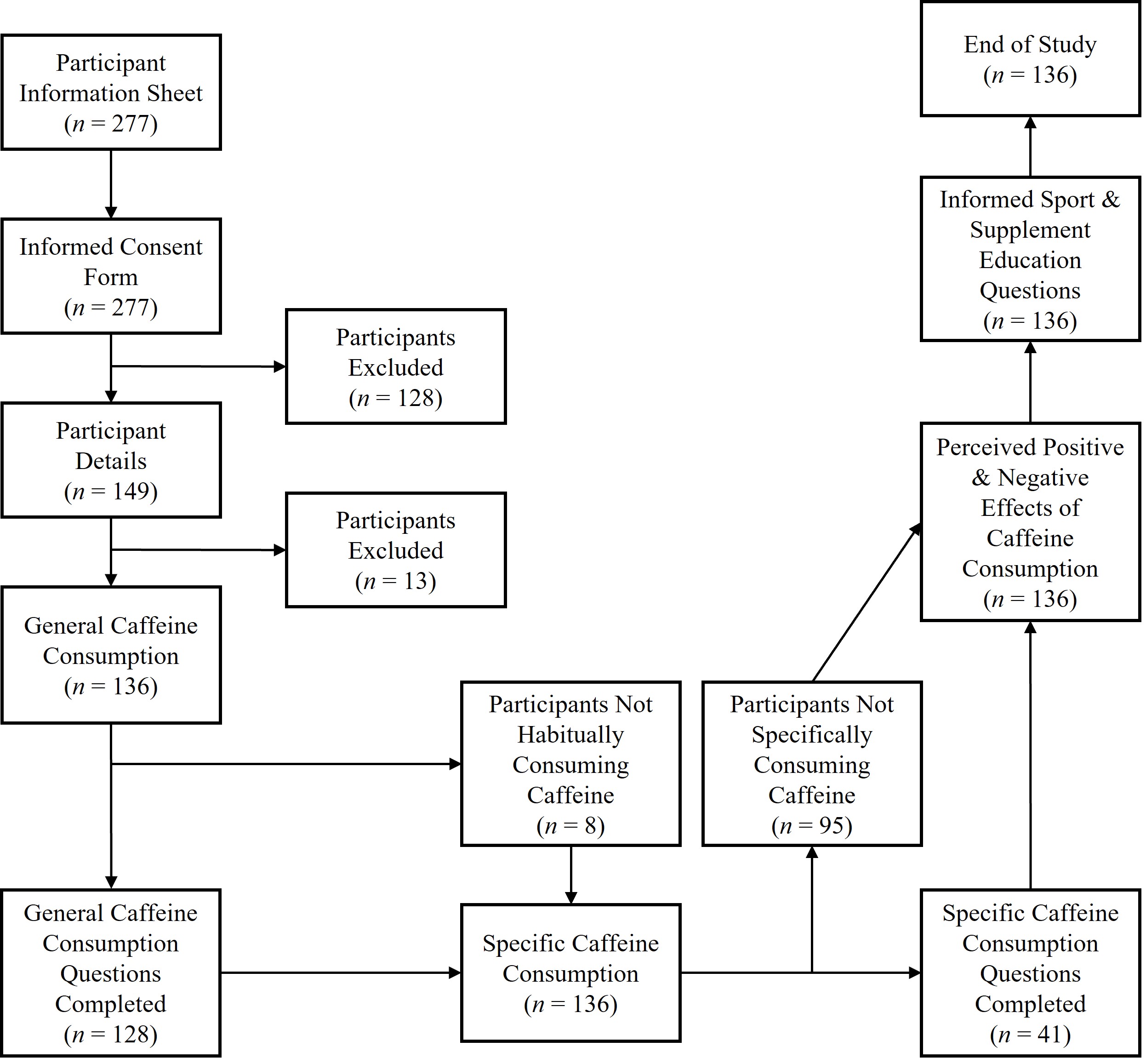

Supplement: Supplementary file 3 [file Image_1.JPEG]
